# Understanding the Assumptions Underlying Instrumental Variable Analyses: a Brief Review of Falsification Strategies and Related Tools

**DOI:** 10.1007/s40471-018-0152-1

**Published:** 2018-06-22

**Authors:** Jeremy Labrecque, Sonja A. Swanson

**Affiliations:** 1 Department of Epidemiology, Erasmus MC, P.O. Box 2040, 3000 CA Rotterdam, The Netherlands; 2 Department of Epidemiology, Harvard T. H. Chan School of Public Health, Boston, MA USA

**Keywords:** Instrumental variable, Falsification, Mendelian randomization

## Abstract

**Purpose of Review:**

Instrumental variable (IV) methods continue to be applied to questions ranging from genetic to social epidemiology. In the epidemiologic literature, discussion of whether the assumptions underlying IV analyses hold is often limited to only certain assumptions and even then, arguments are mostly made using subject matter knowledge. To complement subject matter knowledge, there exist a variety of falsification strategies and other tools for weighing the plausibility of the assumptions underlying IV analyses.

**Recent Findings:**

There are many tools that can refute the IV assumptions or help estimate the magnitude or direction of possible bias if the conditions do not hold perfectly. Many of these tools, including both recently developed strategies and strategies described decades ago, are underused or only used in specific applications of IV methods in epidemiology.

**Summary:**

Although estimating causal effects with IV analyses relies on unverifiable assumptions, the assumptions can sometimes be refuted. We suggest that the epidemiologists using IV analyses employ all the falsification strategies that apply to their research question in order to avoid settings that demonstrably violate a core condition for valid inference.

## Introduction

Many epidemiologists rely on, but are simultaneously skeptical of, the exchangeability (or no uncontrolled confounding) condition required to identify causal effects in our typical analyses of observational studies. Exchangeability is difficult to achieve and impossible to verify, which has led some epidemiologists to prefer instrumental variable (IV) methods that trade in this exchangeability condition for other conditions that are perceived as more plausible in some settings.

IV analysis requires first and foremost an instrumental variable. That is, it requires a variable that meets three conditions: (1) it is associated with the exposure (“relevance”), (2) it only affects the outcome through the exposure (“exclusion restriction”), and (3) its effect on the outcome is unconfounded (“exchangeability”) [1].^1^ These are the three requisite IV conditions, although as we describe below, additional conditions are necessary to identify causal effects. We can see then that, similar to the exchangeability condition in traditional epidemiologic approaches, IV analysis relies on unverifiable conditions. As IV analyses have grown in popularity in recent decades, so have concerns over the plausibility of these assumptions. The most common method of arguing that the IV assumptions hold is using substantive knowledge. However, there are many methods and tools that use the data in hand that can further strengthen or refute the IV assumptions or help estimate the magnitude or direction of possible bias if the conditions do not hold perfectly. Many of these methods are underused or only used in specific applications of IV methods in epidemiology [3••].

Here, we aim to describe and discuss the tools that are available to epidemiologists to strengthen IV analyses (Table 1). Before continuing, we note that IV methods have generally been applied by epidemiologists in four settings: Mendelian randomization studies that propose genetic variants as instruments [18]; pharmacoepidemiologic studies that propose geographic, provider, or temporal variations in prescribing practices as instruments [19]; social epidemiology that propose geographic or temporal variation in policies as instruments [20]; and per-protocol analyses of randomized trials that propose random assignment as an instrument [21]. Each of these settings has unique challenges, but more often than not, the lessons learned from or tools developed within one of these settings could be translated to the others. Given this, our discussion attempts to span all four settings.

**Table 1:** Summary of falsification strategies and related tools for assessing the core conditions for an instrumental variable analysis

Conditions	Strategy	Reference	Restrictions on the settings in which the strategy is applicable
(1)	Check association between instrument and exposure		N/A
(2), (3), (4h)	Over-identification	[4]	Multiple proposed instruments
(2), (3)	Leveraging positive confounding	[5]	Requires knowledge of the direction of confounding
(3)	Negative controls	[6•]	Requires knowledge of the existence of an appropriate negative control
(2)	MR-Egger	[7•]	Multiple proposed instruments; requires additional assumptions*
(2), (3)	Check in a subgroup where the instrument does not work	[8, 9]	Requires knowledge of the existence of such a subgroup
(2), (3)	IV inequalities	[10]	Exposure cannot be continuous
(3)	Covariate balance and bias component plots	[11•]	N/A
(4h)	Checking for differences in instrument strength across covariates	[12]	N/A
(4h)	Estimate counterfactual values among “always-takers,” “compliers,” and “never-takers”	[13]	Condition (4m) must hold and the proposed instrument must be causal
(4m)	Cumulative distribution graphs	[14]	Exposure must be continuous
(4m)	Monotonicity inequalities	[10, 15, 16]	Causal binary proposed instrument, binary exposure, binary outcome
(4m)	Survey of provider preferences	[17]	Proposed instrument must be preference

*See text for further description of the additional assumptions

For the purposes of this review, we always assume the goal is to obtain a numeric estimate for an average causal effect of a treatment or exposure on an outcome. Investigators using IV methods sometimes have other goals, including bounding causal effects or testing causal null hypotheses; reviews of these topics can be found elsewhere [2•].

## Condition (1): Relevance

The first and only verifiable condition is that the proposed instrument must be associated with the exposure. Verifying this only requires checking whether there is an association between the proposed instrument and the exposure. The proposed instrument does not need to cause the exposure but proxy instruments (i.e., instruments that are correlated with but do not cause the exposure themselves) can complicate the interpretation of effect estimates, as we will explain later [22, 23].

Although the relevance condition (1) only requires an association exists, weak associations can mean that the analysis is vulnerable to weak instrument bias either via finite-sample limitations or by amplifying biases due to violations of other assumptions [24]. Therefore, strong instruments are generally preferred over weak instruments. However, deciding whether to use a proposed instrument (or choosing between multiple proposed instruments) solely based on strength, for instance using the *F* statistic or *R*^2^, can also lead to bias because these estimates are more likely to be overestimates of the true instrument strength [25]. These measures of a proposed instrument’s strength are also less directly relevant for understanding whether and how a weak association between the proposed instrument and exposure would result in amplifying biases due to violations of other assumptions.

## Condition (2): Exclusion Restriction

The exclusion restriction condition (2) requires that any effect of the proposed instrument on the outcome is exclusively through its potential effect on exposure. This assumption is not verifiable. Therefore, substantive knowledge of the relationship between the proposed instrument, the exposure and the outcome must be used to justify the plausibility of the exclusion restriction.

There exist, however, methods of falsifying the exclusion restriction with the data, meaning that it is sometimes possible to detect that the assumption is violated (but we cannot ever confirm that it holds). More specifically, many of the available falsification tests jointly test condition (2) alongside condition (3) discussed below. However, because there are settings where condition (3) is expected to hold by design, these falsification strategies are sometimes described as applying to condition (2) alone, which can aid interpretation of the reason for or degree of violation. For example, conditions (2)–(3) jointly can be tested using the instrumental inequalities [10], which can be applied in many settings and can be easily implemented as a one-sided test of a 2 × 2 table in the setting of a binary proposed instrument, binary exposure, and binary outcome [26]. In the all-binary setting and assuming condition (3) holds, a detected violation of the instrumental inequalities also provides some information on the subset of the study population the violation of condition (2) occurs [27].

Other falsification strategies require leveraging additional substantive knowledge. When it is known, for example, that a subgroup can be identified in which the proposed instrument does not affect the exposure, any estimated association between proposed instrument and outcome must be due to a violation of condition (2) or (3) [8, 9]. For example, Kang and colleagues [9] check whether a genetic instrument for malaria has an effect on their outcomes of interest in countries where malaria does not occur. In such places, the genetic instrument cannot have an effect on malaria and any relationship with the outcome must be due to a violation of condition (2) or (3). Assuming that the bias-causing mechanism is homogeneous across subgroups and that the choice of subgroup does not induce selection bias, the bias measured in this subgroup can be used to correct for the violation in the entire population [28•]. Another falsification strategy can be used if the confounding between the exposure and the outcome is known to be positive (i.e., the confounded estimate is larger in magnitude than the true causal effect). This knowledge implies specific relationships between the proposed instrument, exposure, and outcome that can be checked with the data [5].

## Condition (3): Exchangeability

In essence, the exchangeability condition (3) takes the usual exchangeability assumption but forces us to consider exchangeability for the proposed instrument rather than the exposure. Why then, if we are so worried about the exchangeability assumption in traditional analyses, do we find it more plausible in IV analyses? Proposed instruments are often selected because there are a priori reasons to believe that they are exchangeable with respect to the outcome. For instance, if conducting an IV analysis in a randomized trial or lottery study with random assignment as the proposed instrument, exchangeability is expected at baseline by design. In other settings, this assumption must be argued to hold through subject matter knowledge.

**Fig. 1:**
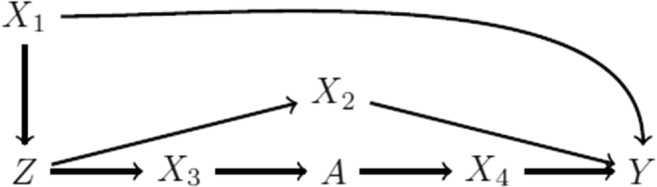
Causal directed acyclic graph of a proposed instrument *Z*, exposure *A*, outcome *Y*, and four additional covariates *X*_1_, *X*_2_, *X*_3_, and *X*_4_. By faithfulness, we would expect that *Z* would be associated with *X*_1_, *X*_2_, *X*_3_, and *X*_4_; however, only associations with *X*_1_ and *X*_2_ indicate violations of the instrumental conditions. Additional unmeasured shared causes of variables in this graph are omitted to simplify presentation

One indirect way of assessing this assumption is to look at whether there is imbalance in measured covariates across levels of the proposed instrument, similar to covariate balance checks in randomized controlled trials. Imbalance in measured covariates can in principle be eliminated by adjusting for them in the analysis, but such imbalances can be suggestive of imbalances across unmeasured variables. As with covariate balance checks in general, an understanding of the causal structure is needed to know whether any perceived imbalances are potentially indicative of bias. In the causal diagram in Fig. 1, *X*_1_, *X*_2_, *X*_3_, and *X*_4_ are expected to be associated with the proposed instrument, but only imbalances in *X*_1_ and *X*_2_ reflect violations of the instrumental conditions (conditions (3) and (2), respectively). Further, of course it is possible that measured covariates appear balanced across levels of the proposed instrument but that there is a lack of exchangeability due to unmeasured covariates: that is, again, we see that we cannot verify condition (3) but may sometimes find suggestive evidence against it. Finally, because the bias due to a violation of condition (3) is a function of the proposed instrument’s strength, it has been argued that these types of covariate balance checks should be augmented to incorporate the proposed instrument’s strength into presentation, especially if presented in conjunction with a non-IV analysis [11•].

Another indirect way of assessing this assumption is to look at negative outcomes, similar to negative outcome controls used in non-IV studies [6•, 29]. Of course, such assessment requires the availability of a secondary outcome that is not expected to be affected by the exposure (or the proposed instrument) but could suffer from the same type of violation of condition (3) that the investigators are concerned about for the primary research question.

## Estimating the Average Treatment Effect with Condition (4h): Homogeneity

In order to obtain a point estimate, a fourth assumption is required and the choice of assumption determines the causal parameter of interest. We first consider the condition (4h) under which the average causal effect is identified.

The homogeneity assumption underlying the standard IV estimator requires that the proposed instrument does not modify the effect of the exposure on the outcome among the exposed and unexposed on the additive scale. If any unmeasured confounder of the exposure-outcome relationship is also an effect measure modifier, then it is usually not reasonable to assume condition (4h) homogeneity [23]. Some investigators propose conditioning on measured covariates that are perceived to be important effect measure modifiers to recover the average causal effect [30, 31].

Assessment of the homogeneity conditions remains difficult, and discussions of this condition remain complicated in the literature because debates remain on how prevalent relevant heterogeneity is in epidemiology [32]. (Of course, this varies depending on the study question.) Some relatively simple checks have been proposed to falsify or understand the importance of condition (4h) in a particular study. For example, conditions (1)–(3) alone allow for bounding of the average causal effect [10, 15, 16], which means that when the bounds achieved under these three conditions alone are wide, then it is at least mathematically possible for the point estimate to be very different from the true causal effect due to a violation of condition (4h).

In the simple setting of a dichotomous source of effect measure modification, Brookhart et al. [12] showed that the bias due to a violation of condition (4h) is a function of how the strength of the proposed instrument differs within strata of the modifier. Given this, the investigators proposed presenting how the strength of the proposed instrument differs across measured covariates. The logic here is similar to that of presenting covariate balance when considering condition (3): any detected differences in measured covariates could theoretically be accounted for by including the modifier in the model, but may arguably indicate that there are unmeasured sources of effect measure modification that violate condition (4h).

When both the proposed instrument and exposure are binary, and the proposed instrument causes the exposure, then one can compare the counterfactual outcomes between “compliance types” as an indirect assessment of condition (4h) [13]. A study participant’s membership in one of the four mutually exclusive compliance types is determined by how that person is affected by the instrument: people who are exposed regardless of the instrument (“always-takers”), people who are never exposed regardless of the instrument (“never-takers”), people who are coerced to be exposed because of the instrument (“compliers”), and people who do the opposite of “compliers” (“defiers”). When there are no “defiers,” it is possible to estimate the counterfactual outcome of both “never-takers” and “compliers” under no exposure from the data. If these values are different, it is calls the homogeneity assumption into question. The same can be done with the counterfactual outcomes of “always-takers” and “compliers” under exposure.

Finally, while the usual IV estimators tend to assume additive effect homogeneity, there is another IV estimator based on a multiplicative rather than additive structural mean model that relies on a similar assumption but on the multiplicative scale [15, 23, 33]. As homogeneity cannot simultaneously be satisfied on both scales except under the null, careful thought should be given to whether one of these two scales and therefore one of these two types of conditions (4h) is more likely to hold.

## Estimating the Local Average Treatment Effect with Condition (4m): Monotonicity

Economists, who are responsible for developing much of the early theory of IV methods, have generally been skeptical of the homogeneity condition (4h) and instead looked toward a monotonicity condition (4m) as a sometimes more plausible, alternative assumption. In the usual way that condition (4m) is evoked, monotonicity requires that the proposed instrument only affects the exposure in one direction in all individuals. In other words, there do not exist both people whose exposure level would have been increased by increases in the proposed instrument and people whose exposure level would have been decreased by increases in the proposed instrument. (Recently, different versions of monotonicity conditions have been described that can change the interpretation of the effect estimate, but go beyond the purposes of this review [17, 34].) If monotonicity is assumed instead of homogeneity, an average causal effect in the subgroup of “compliers” (described above) is identified [35–37]. Of note, some investigators have argued against estimating this effect because the subgroup of “compliers” is not identified, and for proxy or non-binary proposed instruments, the interpretation of this subgroup becomes even less clear [22, 23, 38]. The non-identifiability concern is partially mitigated when the proposed instrument and exposure are binary and the proposed instrument is causal: then conditions (1)–(3) and (4m) allow us to estimate the proportion of “compliers” as well as describe their characteristics in measured covariates [39]. When the proposed instrument is a proxy (i.e., non-causal) instrument, estimating the proportion of or characterizing the “compliers” becomes more difficult and requires additional assumptions and considerations of the underlying causal instrument [22, 23].

When the exposure is continuous, violations of condition (4m) can be found by graphing the difference in cumulative distribution in exposure for each level of the proposed instrument [14]. If the difference in cumulative distribution functions changes sign over the range of feasible exposure levels, then a violation of the monotonicity assumption is detected. Failure to detect a violation, however, does not constitute support for the monotonicity assumption.

When the proposed instrument is a measure of a decision-maker’s preference, such as the commonly proposed provider preference instruments in pharmacoepidemiology studies, then it is also possible to empirically assess the monotonicity condition (4m) by supplementing the data with a survey of the providers [17]. By asking providers about their treatment decisions for the same set of (possibly hypothetical) patients, investigators can measure the compliance type distribution and potentially find evidence against monotonicity.

Finally, relatively simple inequalities can be checked to falsify monotonicity in the simple case of a binary proposed causal instrument, binary exposure, and binary outcome (assuming conditions (1), (2), and (3) hold). Coincidentally, these inequalities will be violated whenever the bounds on the average causal effect proposed by Manski and Robins differ from those proposed by Balke and Pearl [10, 15, 16]; see reference [2•] for more detail.

## Settings with Multiple Proposed IVs

In some studies, investigators suggest that there is not just one but multiple proposed instruments. In such settings, it is sometimes possible to leverage this added potential information in ways that relax the instrumental conditions or that mitigate bias due to some of the proposed instruments not being instruments. For a more complete review of the available sensitivity analyses and robust methods, see reference [40••]. We briefly highlight how having multiple proposed instruments can potentially address or detect some of the biases due to violations of the above-described conditions for each proposed instrument.

First, having multiple proposed instruments affords more ways to conduct an IV analysis. Investigators proposing multiple instruments have historically estimated causal effects by meta-analyzing individual estimates, by using all proposed instruments in the same two-stage least squares regression models, and by combining the proposed instruments into a summary risk score [41]. Generally, these methods can be more robust to weak instrument biases, although it is important to be aware of the assumptions made in each case. Newer estimators, primarily developed in the Mendelian randomization literature, can also allow for some types of violations of the exclusion restriction condition (2) [7•, 42, 43] by leveraging additional assumptions (for a complete review of these additional assumptions, see reference [40••]). MR-Egger in particular allows for certain violations of condition (2) by introducing homogeneity and linearity assumptions alongside the assumption that the strength of each proposed instrument is independent of the size of the direct effect violating condition (2); under these assumptions, MR-Egger can also be used to detect the existence of these types of violations of condition (2) for some of the proposed instruments [7•].

Another oft-used falsification strategy in the multiple instrument setting is the Hausman over-identification test in which all proposed instruments are regressed on the residuals of a regression of the outcome on the exposure. It can identify whether at least one of the proposed instruments used is invalid but cannot identify which [4]. Moreover, it cannot detect which condition is violated among conditions (2), (3), or (4h). Such a test is especially underpowered and may not valid for weak instruments [44].

Importantly, because any sound method for estimating a causal effect or falsifying assumptions via multiple proposed instruments needs to be targeting the same average causal effect (rather than the instrument-specific causal effects within the “compliers”), these approaches all rely on a homogeneity condition (4h) or even stronger homogeneity or linearity conditions. This means that using multiple proposed instruments in settings where effect heterogeneity is likely (i.e., in which condition (4h) is unlikely to hold) makes interpreting effect estimates nearly impossible [45].

## Beyond the Core IV Conditions

We have discussed the core assumptions that IV analyses make in replace of the usual exchangeability condition underlying non-IV analyses. However, there are some additional considerations worth noting. First, the core above-described assumptions replace our more typical assumption of no uncontrolled baseline confounding, but this means that any selection or information biases that could affect a non-IV analysis in the same study may also be problematic for an IV analysis. In addition to the more familiar selection biases (e.g., due to loss to follow-up [46, 47]), subtler selection biases can arise in IV analyses that select on a subset of possible exposures [48, 49]. Also, it is of course possible if not likely that in many observational studies with proposed instruments, the investigators may believe the above-described assumptions do not hold unconditionally but are more reasonable within levels of measured covariates; in such cases, investigators may consider applying the falsification strategies and tools described here within levels of the measured covariates.

Importantly, estimating an average causal effect in an IV or non-IV analysis alike requires having an unambiguous definition of the intervention regardless of the estimation procedure used [50, 51]. In some studies employing IV analyses, such as with randomized trials or program evaluation, the intervention is well-defined because it has been actually implemented. However, in some studies employing IV analyses, such as many Mendelian randomization studies, the intervention itself is not well-defined [52•]. This makes it very difficult to interpret or assess the validity of any presented effect estimates. Such vagueness also often overlooks the fact that classical IV methods are developed in the context of time-fixed treatments, and thus when the exposures can vary over time, it is both less clear what investigators are trying to estimate and why the above-described assumptions are reasonable [52•, 53, 54•] .

Finally, up until now, our consideration of falsification strategies and related tools has focused on understanding the validity of an IV analysis. Sometimes, IV analyses are performed alongside non-IV analyses, and investigators are interested in understanding whether the IV analysis is more or less biased than the non-IV analysis. Bias component plots have been proposed as one option for considering relative bias due to violations in exchangeability across IV and non-IV methods [11•]. Investigators also sometimes begin by comparing the estimates from the two approaches, either using subjective criteria or a formal test [4]. However, any detected differences could mean that the IV analysis is biased, the non-IV analysis is biased, the analyses are estimating different causal effects (e.g., the effect in the “compliers” vs. the average causal effect), or all of the above.

## Conclusion

Addressing a causal question with methods that make different assumptions demonstrates whether estimates are sensitive to the assumptions that differ across methods. In this way, estimates obtained from IV analyses can nicely complement estimates from analyses that require measuring and appropriately adjusting for confounders. However, the plausibility of these and any conditions required for causal interpretations must still be verified when possible and, when verification is not possible, efforts must be made to falsify the conditions as feasible. Here we have assembled a list of techniques that can be used to falsify or weigh the reasonableness of the core conditions underlying IV analyses. By using all techniques applicable to a specific epidemiologic IV analysis, we can use the data to its fullest extent.

## Footnotes

1 The IV conditions have been formalized a number of ways, but for the purposes of this review the primary differences between these formalizations are not usually relevant. For more information on the various formalizations, see reference [2•].
